# OLOPATADINE IN CHRONIC IDIOPATHIC URTICARIA

**DOI:** 10.4103/0019-5154.53180

**Published:** 2009

**Authors:** Kiran V Godse

**Affiliations:** *From the Shree Skin Centre, 22, L Market, Sector 8, Nerul, Navi Mumbai - 400 706, India. E-mail: drgodse@yahoo.co.in*

Sir,

Olopatadine hydrochloride is one of the second-generation nonsedating antihistamines that is used for treating allergic disorders such as urticaria, rhinitis, and atopic dermatitis.[1] Olopatadine is a selective histamineH1-receptor antagonist possessing inhibitory effects on the release of inflammatory lipid mediators, such as leukotriene and thromboxane from human polymorphonuclear leukocytes and eosinophils.[2] Olopatadine was shown to be useful for the treatment of allergic rhinitis and chronic urticaria in double-blind clinical trials. Olopatadine was approved in Japan for the treatment of allergic rhinitis, chronic urticaria, eczema dermatitis, prurigo, pruritis cutaneous, psoriasis vulgaris, and erythema exsudativum multiforme in December 2000.[2]

Olopatadine hydrochloride was tested in patients with chronic idiopathic urticaria (CIU). CIU is defined by the almost daily presence of urticaria for at least six weeks without an identifiable cause. Aim of this study was to investigate the efficacy and side effects of olopatadine hydrochloride in CIU patients.

Twenty CIU patients in the age range of 14–70 years (11 men and 9 women, mean age: 41.95 years) were included in the study [Table 1]. Duration of urticaria ranged from 8–156 weeks (3 years) with average duration of 52 weeks (1 year) [Table 2]. History of NSAID allergy was present in two patients. Physical urticaria patients, pregnant women, and lactating mothers were not included in the study. All patients were investigated thoroughly with general and systemic examinations to rule out any septic focus or any obvious cause of urticaria, and their complete blood count, urine, and glucose levels were analyzed before initiating the therapy. Informed consents from all the study participants were obtained at their initial visit. All medications including antihistaminics were stopped a day prior to the study. The treatment comprised of T. olopatadine 5 mg orally twice a day after meals. The duration of therapy lasted either till the symptoms alleviated or up to one month. The clinical efficacy was measured using the urticaria activity score (UAS) at each follow-up. The follow-up evaluation was at 0, 2, and 4 weeks of therapy initiation. UAS is the sum of wheal number score and itch severity score.[3] The wheal numbers are graded from 0–3 as follows: 0 = <10 small wheals (diameter, <3 cm); 1 = 10–50 small wheals or <10 large wheals (diameter, >3 cm); 2 = >50 small wheals or 10–50 large wheals; and 3 = almost the whole body is covered. The severity of itching is graded from 0–3 (0, none; 1, mild; 2, moderate; and 3, severe).

Average UAS was 3.7 at 0 weeks which went down to 1.95 at the end of two weeks. At the end of four weeks, UAS further lessened to 1.2. Difference in UAS at 0 week and at 4 weeks was statistically significant (*P*<0.05). Two patients (10%) complained of headache and sedation after initiation of the therapy. Of eight patients (6 men and 2 women) with more than 36 weeks of urticaria, one patient did not respond to therapy and administration of steroid became necessary to control the condition. Olopatadine therapy was well tolerated without major side effects by most patients in this study.

Olopatadine, a newly developed histamine H1-receptor antagonist, was compared with cetirizine in its suppressive effects on histamine-induced wheal and flare reaction using an iontophoresis technique in a double-blind, cross-over, placebo-controlled study. As a result, olopatadine was found to have effects comparable to cetirizine.[4] In another study olopatadine and cetirizine showed the strongest and most persistent suppression of histamine-induced wheal and flare response. Also, olopatadine showed a considerable sedative effect with impaired psychomotor performance.[5]

Olopatadine is an effective treatment for CIU with sedation being a side effect in 10% of patients. Further studies in this direction with larger study groups are required to vindicate above findings.

**Table 1:** Age range of the study population

Age group (years)	Men	Women
18–35	3	4
36–50	3	3
51–70	5	2

**Table 2:** Duration of urticaria

Duration of urticaria (weeks)	Men	Women
6–36	5	7
37–156	6	2
